# The relationships of preventive behaviors and psychological resilience with depression, anxiety, and stress among university students during the COVID-19 pandemic: A two-wave longitudinal study in Shandong Province, China

**DOI:** 10.3389/fpubh.2023.1078744

**Published:** 2023-03-21

**Authors:** Hexian Li, Jingjing Zhao, Rui Chen, Hui Liu, Xixing Xu, Jing Xu, Xiaoxu Jiang, Mingli Pang, Jieru Wang, Shixue Li, Jiaxiang Hou, Fanlei Kong

**Affiliations:** ^1^Center for Health Management and Policy Research, School of Public Health, Cheeloo College of Medicine, Shandong University, Jinan, China; ^2^NHC Key Lab of Health Economics and Policy Research, Shandong University, Jinan, China; ^3^School of Marxism, Shandong University, Jinan, China; ^4^Shandong Center for Disease Control and Prevention, and Academy of Preventive Medicine, Shandong University, Jinan, China

**Keywords:** COVID-19, college students, depression, anxiety, stress, resilience, longitudinal study

## Abstract

**Introduction:**

Studies have shown that the psychological impact of the COVID-19 pandemic may lead to long-term health problems; therefore, more attention should be paid to the mental health of university students. This study aimed to explore the longitudinal effects of preventive behaviors and psychological resilience on the mental health of Chinese college students during COVID-19.

**Methods:**

We recruited 2,948 university students from five universities in Shandong Province. We used a generalized estimating equation (GEE) model to estimate the impact of preventive behaviors and psychological resilience on mental health.

**Results:**

In the follow-up survey, the prevalence of anxiety (44.8% at T1 vs 41.2% at T2) and stress (23.0% at T1 vs 19.6% at T2) decreased over time, whereas the prevalence of depression (35.2% at T1 vs 36.9% at T2) increased significantly (*P* < 0.001). Senior students were more likely to report depression (OR = 1.710, *P* < 0.001), anxiety (OR = 0.815, *P* = 0.019), and stress (OR = 1.385, *P* = 0.011). Among all majors, medical students were most likely to report depression (OR = 1.373, *P* = 0.021), anxiety (OR = 1.310, *P* = 0.040), and stress (OR = 1.775, P < 0.001). Students who wore a mask outside were less likely to report depression (OR = 0.761, *P* = 0.027) and anxiety (OR = 0.686, *P* = 0.002) compared to those who did not wear masks. Students who complied with the standard hand-washing technique were less likely to report depression (OR = 0.628, *P* < 0.001), anxiety (OR = 0.701, *P* < 0.001), and stress (OR = 0.638, *P* < 0.001). Students who maintained a distance of one meter in queues were less likely to report depression (OR = 0.668, *P* < 0.001), anxiety (OR = 0.634, *P* < 0.001), and stress (OR = 0.638, *P* < 0.001). Psychological resilience was a protective factor against depression (OR = 0.973, *P* < 0.001), anxiety (OR = 0.980, *P* < 0.001), and stress (OR = 0.976, *P* < 0.001).

**Discussion:**

The prevalence of depression among university students increased at follow-up, while the prevalence of anxiety and stress decreased. Senior students and medical students are vulnerable groups. University students should continue to follow relevant preventive behaviors to protect their mental health. Improving psychological resilience may help maintain and promote university students' mental health.

## 1. Introduction

Historically, humans have experienced various health emergencies caused by pandemics and epidemics worldwide. By 30 January 2020, the WHO had declared the COVID-19 outbreak to be a public health emergency of international concern (1). To efficiently address the COVID-19 outbreak, the Chinese government implemented rapid and comprehensive public health emergency interventions. From 24 January 2020, all 31 provincial-level regions in China with confirmed COVID-19 cases activated a Level 1 public health emergency response (2). To prevent the spread of the disease, the Chinese Government delayed the re-opening of all schools, including universities. Therefore, university students in China have been required to stay at home, in isolation, for longer periods of time, which may increase their risk of depression (3). Previous studies have shown that the COVID-19 pandemic has exerted negative psychological effects on people (4–6). Several cross-sectional studies have shown that Chinese university students experienced varying degrees of mental health problems during COVID-19 (7–9). One study has suggested that the psychological impact of the pandemic may lead to long-term health problems (10). Therefore, greater attention should be paid to the mental health of university students.

Wearing a mask (11), maintaining hand hygiene (12), and maintaining physical distance from others (13) during COVID-19 have been recognized as effective pandemic prevention measures and are strongly advocated by the WHO. The Chinese government implemented laws mandating these preventive behaviors (14, 15). Studies have found that engaging in preventive behaviors affects mental health. University students in Ethiopia who engaged in poor prevention practices were twice as likely to experience psychological problems due to COVID-19 (16). A study by Green et al. with university students in Pakistan showed that higher levels of fear of COVID-19 were linked to greater adherence to COVID-19 preventive behaviors (17). However, Ikram et al. showed that wearing a mask was a predictor of poor mental health among Asian Indians (18). In contrast, Abir et al. found that Bangladeshis who did not wear masks and who did not comply with WHO precautions were more likely to report psychological ill health (19). Guan et al. (20) found that positive preventive behaviors showed protective effects against anxiety among Chinese university students (20).

In the context of the COVID-19 pandemic, psychological resilience may be a potential factor affecting university students' mental health. Psychological resilience is the process and outcome of successful adaptation to difficult or challenging life experiences (21). A high level of resilience protects against various mental health conditions (22). Previous studies have shown that psychological resilience has been negatively correlated with depression, anxiety, and stress during COVID-19 (23–25). A study of young adults in Turkey during COVID-19 showed that resilience mediated the effect of finding meaning in life on young people's psychological, emotional, and social wellbeing. Therefore, resilience might modify the adverse effect of the coronavirus pandemic on young people's mental wellbeing (26). A study of Korean adults by Kim et al. showed that individual resilience had an effect on mental health after the COVID-19 outbreak (27). Additionally, a study conducted in Minnesota and Hong Kong found that individual resilience was associated with positive mental health during COVID-19 (28). Azizah et al. (29) found that lower levels of psychological resilience during the COVID-19 pandemic were associated with increased levels of depression, anxiety, and stress among university students (29). Similarly, Tan et al. (30) reported that psychological resilience had a strong positive effect on university students' mental health during the pandemic (30).

**Figure 1:**
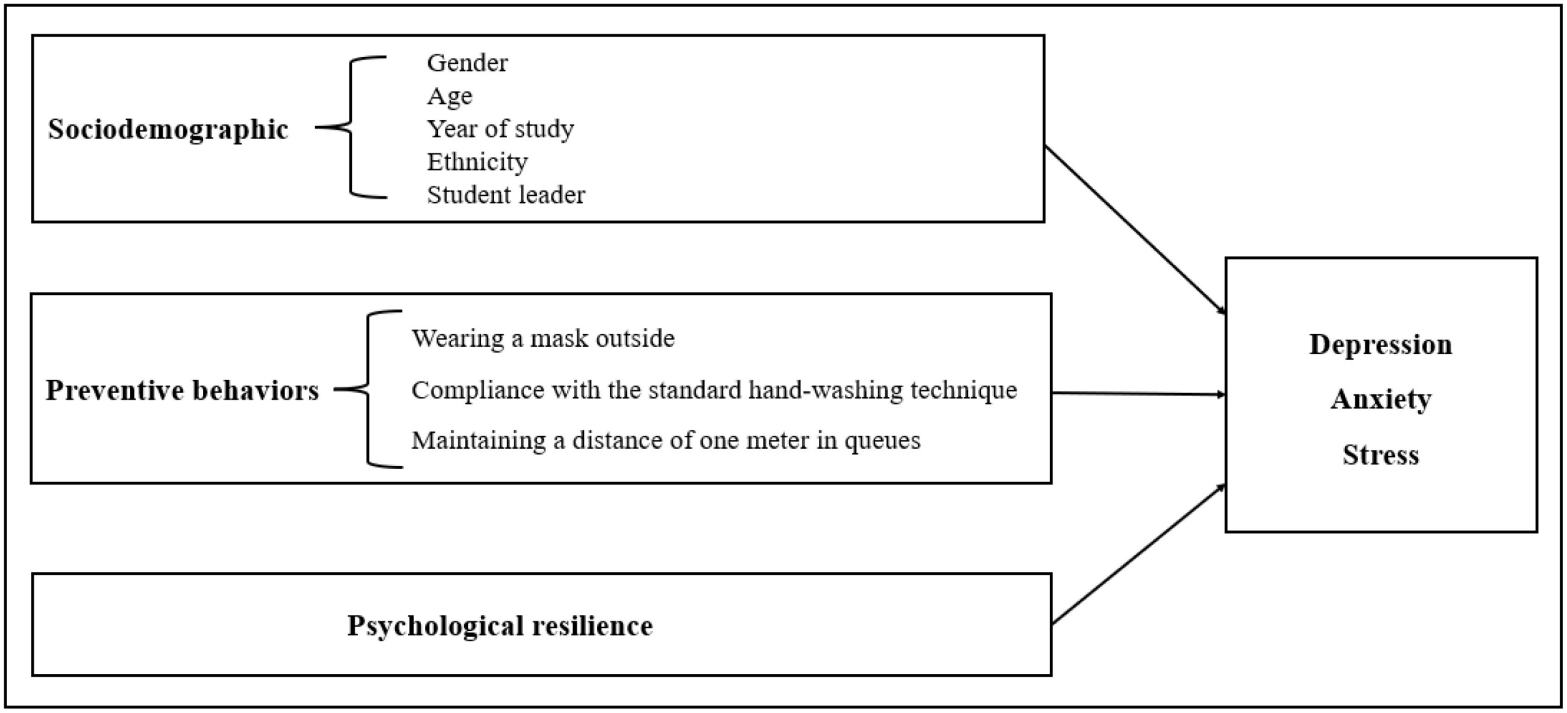
The conceptual model of this study.

Most extant studies on the mental health of university students during COVID-19 have employed cross-sectional designs (31–33), with few studies conducting longitudinal investigations. Theoretically, existing research has shown that individual psychological factors and behaviors could affect health (34). However, to date, no study has longitudinally examined the relationship between preventive behaviors, psychological resilience, and mental health (depression, anxiety, and stress). Thus, this study explored longitudinal changes in mental health and the longitudinal effects of preventive behaviors and psychological resilience on the mental health of university students in Shandong Province, China during COVID-19 (as shown in the conceptual model in Figure 1). We conducted a large-scale longitudinal survey and followed up with the same population of students before and after the winter holiday and COVID-19 vaccination. We aimed to identify trends in the development of depression, anxiety, and stress to accomplish the abovementioned research purposes, and to provide stronger statistical evidence than can be obtained from cross-sectional data.

## 2. Methods

### 2.1. Study design and participants

This prospective study was conducted at five universities in Shandong Province. Various majors (such as art, science, medicine, and agriculture) offered at the five universities were selected via stratified random sampling according to geographical location (east, middle, or west) and college category (key or general). Students from one class were randomly selected from each year of each selected major to participate.

A total of 4,832 students (valid response rate: 82%) completed the initial survey between 20 October 2020 and 6 November 2020 (T1); participants were on-campus, non-graduating students who were returning to campus after having studied under the pandemic-preventive order for approximately two months. During the second wave of the survey from 18 January to 25 January 2021 (T2), 4,408 students (valid response rate: 91.54%) completed the survey. During this period, the Chinese government began to provide COVID-19 vaccines for all citizens; this was also the time at which students returned home for the winter vacation after completing their autumn term of study on campus. Using the student numbers of the university students to match initial and follow-up responses, a total of 2,948 students were found to have participated in both the T1 and T2 surveys and were selected as our study participants.

### 2.2. Procedures

Due to limited resources and the implementation of social distancing, the survey was completed through the China Survey Star website. Informed consent was obtained from every student who participated in the study. Full-time counselors organized the administrative processes for both waves, and electronic questionnaires were distributed to class groups, where students were asked to fill them out within a specified time frame. Only one response was permitted from any IP address. For the second wave, counselors followed up with the students *via* WeChat and phone. After the data were collected, invalid sets of responses were removed, as identified by one of the three following criteria being met: (1) the response time was <120 s; (2) information on sociodemographic characteristics was missing or not relevant to the survey; or (3) the responses were illogical.

### 2.3. Measurement

#### 2.3.1. Sociodemographic characteristics

The sociodemographic characteristics measured included gender (male vs. female), age, year of university study (1st year, 2nd year, 3rd year, or 4th/5th year), major (science, engineering, agriculture, liberal arts, art, or medicine), ethnicity (Han Chinese vs. minority), and student leader (yes or no).

#### 2.3.2. Preventive behaviors

According to the WHO, preventive behaviors for COVID-19 include hand-washing, wearing a mask, and maintaining social distancing (35). Preventive behaviors were measured using the following questions: “Have you been wearing a mask outside in the past 2 weeks?” (yes/no), “Have you followed standard hand-washing techniques in the last 2 weeks?” (yes/no), and “Have you been standing one meter apart in line for the last 2 weeks?” (yes/no).

#### 2.3.3. Depression anxiety stress scale

The DASS-21 was used to assess participants' levels of depression, anxiety, and stress. The scale consists of seven items on each dimension (depression, anxiety, and stress). Response options range from 0 to 3 (0 = *did not apply to me at all*, 1 = *applied to me to some degree, or some of the time*, 2 = *applied to me to a considerable degree or a good part of the time*, and 3 = *applied to me very much or most of the time*) (36). Additionally, because the DASS-21 is a short version of the original 42-item DASS instrument, DASS-21 scores were multiplied by 2 to characterize the level of severity relative to the population. Depression severity was classified into five categories: normal (0–9), mild (10–13), moderate (14–20), severe (21–27), and very severe (28+). Similarly, for anxiety, the categories were normal (0–7), mild (8–9), moderate (10–14), severe (15–19), and very severe (20+). Stress scores were categorized as normal (0–14), mild (15–18), moderate (19–25), severe (26–33), or very severe (34+) (37). The scores obtained on these three subscales were dichotomized (38). Specifically, students falling in the moderately, severely, and extremely severely depressed, anxious, and stressed categories were considered to be depressed, anxious, and stressed, respectively; others were considered to be not depressed, not anxious, and not stressed, respectively. Cronbach's alpha values in the current study for the depression, anxiety, and stress subscales were 0.915, 0.876, and 0.892, respectively, at T1, and 0.928, 0.901, and 0.917, respectively, at T2, indicating good internal consistency.

#### 2.3.4. Psychological resilience (CD-RISC-10)

Psychological resilience was assessed using the Connor-Davidson Resilience Scale (CD-RISC-10), which consists of 10 items with 5 response options ranging from 0 (*never*) to 4 (*almost always*) (39, 40). The CD-RISC-10 is widely used to assess an individual's perception of their ability to thrive under adversity. Total scores range from 0 to 40, with higher scores indicating greater resilience. The CD-RISC-10 has been shown to have satisfactory validity and reliability in Chinese university students (41). In this study, Cronbach's α was 0.971 at T1 and 0.979 at T2.

#### 2.3.5. Statistical analysis

First, we described the characteristics of participants at TI and T2 and used the chi-square test and paired *t*-tests to examine changes in depression, anxiety, stress, psychological resilience, and related variables for participants at both time points. Second, the longitudinal effect of psychological resilience on depression, anxiety, and stress was analyzed using generalized estimating equations (GEEs), and three models were built to control for confounding variables. Demographic characteristics were included in Model 1; demographic characteristics and pandemic preventive behaviors were included in Model 2; and demographic characteristics, pandemic preventive behaviors, and psychological resilience were included in Model 3. All statistical tests were performed using IBM SPSS 26.0. Statistical significance was set at *P* < 0.05.

## 3. Results

### 3.1. Description of the sample and study variables

A total of 2,948 university students who completed both the baseline and follow-up surveys were included in the analyses. Detailed sample demographics, along with data on preventive behaviors and psychological resilience at both time points, are shown in Table 1. University students who participated at both T1 and T2 were mostly women (63.6%). Participants were aged 19.85 ± 1.449 years at T1 and 20.20 ± 1.521 years at T2. The proportion of students who wore a mask outside was 90.6% at T1 and 97.8% at T2; the rate of compliance with the standard hand-washing technique was 76.8% at T1 and 84.0% at T2; and the proportion of students who maintained a one-meter distance in queues was 60.6% at T1 and 78.1% at T2. In all three cases, the proportion increased at T2. The average psychological resilience score at T2 was 29.22 ± 8.532, which was lower than that at T1 (29.25 ± 8.406; *P* = 0.653). As shown in Table 2, the prevalences of depression, anxiety, and stress at T1 were 35.2, 44.8, and 23.0%, respectively. Rates of anxiety (44.8% at T1 vs. 41.2% at T2) and stress (23.0% at T1 vs. 19.6% at T2) decreased over time, whereas the rate of depression (35.2% at T1 vs. 36.9% at T2) increased significantly (*P* < 0.001).

**Table 1:** Changes in COVID-19-related variables between T1 and T2.

**Variables**	**T1**	**T2**	**χ^2^/*t***	***P***
**Gender**				
Male	1,072 (36.4%)	1,072 (36.4%)		
Female	1,876 (63.6%)	1,876 (63.6%)		
Age	19.85 ± 1.449	20.20 ± 1.521	−9.061^b^	<0.001
**Year of study**				
1st year	762 (25.8%)	762 (25.8%)		
2nd year	661 (22.4%)	661 (22.4%)		
3rd year	796 (27.0%)	796 (27.0%)		
4th/5th year	729 (24.7%)	729 (24.7%)		
**Major**				
Science	502 (17.0%)	502 (17.0%)		
Engineering	830 (28.2%)	830 (28.2%)		
Agriculture	145 (4.9%)	145 (4.9%)		
Liberal arts	1,041 (35.3%)	1,041 (35.3%)		
Art	254 (8.6%)	254 (8.6%)		
Medicine	176 (6.0%)	176 (6.0%)		
**Ethnicity**				
Han	2,834 (96.2%)	2,834 (96.2%)		
Minority	113 (3.8%)	113 (3.8%)		
**Student leader**				
Yes	798 (27.1%)	798 (27.1%)		
No	2,150 (72.9%)	2,150 (72.9%)		
**Wearing a mask outside**				
No	277 (9.4%)	66 (2.2%)	21.228^a^	<0.001
Yes	2,671 (90.6%)	2,882 (97.8%)		
**Compliance with the standard hand-washing technique**				
No	684 (23.2%)	472 (16.0%)	416.307^a^	<0.001
Yes	2,264 (76.8%)	2,476 (84.0%)		
**Maintaining a distance of one meter in queues**				
No	1,161 (39.4%)	646 (21.9%)	340.813^a^	<0.001
Yes	1,787 (60.6%)	2,302 (78.1%)		
Psychological resilience	29.25 ± 8.406	29.22 ± 8.532	0.450^b^	0.653

a Chi-square test,

b t-test.

**Table 2:** Prevalence of and change in depression, anxiety, and stress at T1 and T2.

	**T1**	**T2**	**χ^2^**	***P***
**Depression**				
No (≤9%)	1,909 (64.8%)	1,861 (63.1%)	674.018^a^	<0.001
Yes (≥10%)	1,039 (35.2%)	1,087 (36.9%)		
**Anxiety**				
No (≤7%)	1,628 (55.2%)	1,734 (58.8%)	660.175^a^	<0.001
Yes (≥8%)	1,320 (44.8%)	1,214 (41.2%)		
**Stress**				
No (≤14%)	2,269 (77.0%)	2,370 (80.4%)	428.475^a^	<0.001
Yes (≥15%)	679 (23.0%)	578 (19.6%)		

### 3.2. Association of pandemic preventive behaviors and psychological resilience with depression

The findings of the GEE analysis of depression are presented in Table 3; Model 3 represents the results after controlling for sociodemographic characteristics and preventive behavior. Gender, year of university study, major, preventive behavior-related variables, and psychological resilience were significantly associated with depression. Specifically, women were less likely to report depression than men (OR = 0.686, *P* < 0.001), while students in their 4th or 5th year of university (OR = 1.710, *P* < 0.001), as well as those majoring in liberal arts (OR = 1.238, *P* = 0.012), art (OR = 1.286, *P* = 0.032), or medicine (OR = 1.373, *P* = 0.021), were more likely to report depression over time. Those who wore a mask outside (OR = 0.761, *P* = 0.027), complied with the standard hand-washing technique (OR = 0.628, *P* < 0.001), maintained a distance of one meter in queues (OR = 0.668, *P* < 0.001), and had high psychological resilience scores (OR = 0.973, *P* < 0.001) were less likely to report depression over time.

**Table 3:** Longitudinal associations of epidemic preventive behaviors and psychological resilience with depression based on GEE.

**Variables**	**Model 1**			**Model 2**			**Model 3**		
	**OR**	**95% CI**	***P***	**OR**	**95% CI**	***P***	**OR**	**95% CI**	***P***
**Gender**									
Male	Ref.			Ref.			Ref.		
Female	0.701	0.624, 0.787	<0.001	0.684	0.607,0 0.770	<0.001	0.686	0.609,0.774	<0.001
Age	1.039	1.002, 1.078	0.039	1.040	1.002, 1.079	0.040	1.003	0.966, 1.043	0.862
**Year of study**									
1st year	Ref.			Ref.			Ref.		
2nd year	1.122	0.955, 1.318	0.161	1.121	0.953, 1.320	0.168	1.117	0.948, 1.316	0.187
3rd year	1.376	1.184, 1.599	<0.001	1.333	1.145, 1.552	<0.001	1.332	1.143, 1.551	<0.001
4th/5th year	1.761	1.512, 2.051	<0.001	1.719	1.472, 2.007	<0.001	1.710	1.464, 1.998	<0.001
**Major**									
Science	Ref.			Ref.			Ref.		
Engineering	1.123	0.947, 1.331	0.181	1.164	0.981, 1.381	0.083	1.150	0.968, 1.368	0.112
Agriculture	1.076	0.814, 1.422	0.606	1.079	0.813, 1.431	0.601	1.090	0.820, 1.450	0.552
Liberal arts	1.205	1.024, 1.419	0.025	1.255	1.064, 1.480	0.007	1.243	1.052, 1.469	0.011
Art	1.204	0.961, 1.507	0.106	1.327	1.057, 1.667	0.015	1.285	1.021, 1.618	0.033
Medicine	1.355	1.047, 1.753	0.021	1.382	1.063, 1.797	0.016	1.373	1.055, 1.788	0.018
**Ethnicity**									
Han	Ref.			Ref.			Ref.		
Minority	0.854	0.641, 1.137	0.280	0.894	0.670, 1.194	0.448	0.878	0.658, 1.170	0.373
**Student leader**									
Yes	Ref.			Ref.			Ref.		
No	1.109	0.981, 1.253	0.097	1.092	0.965, 1.236	0.165	1.085	0.957, 1.229	0.201
**Wearing a mask outside**									
No				Ref.			Ref.		
Yes				0.716	0.563, 0.912	0.007	0.761	0.598, 0.969	0.026
**Compliant with the standard hand-washing technique**									
No				Ref.			Ref.		
Yes				0.648	0.555, 0.757	<0.001	0.628	0.538, 0.735	<0.001
**Maintaining a distance of one meter in queues**									
No				Ref.			Ref.		
Yes				0.682	0.595, 0.782	<0.001	0.668	0.582,0 0.766	<0.001
Psychological resilience							0.973	0.966, 0.979	<0.001

### 3.3. Association of pandemic preventive behaviors and psychological resilience with anxiety

The GEE results for anxiety, presented in Table 4, demonstrate that, in Model 3, women were less likely to report anxiety (OR = 0.752, *P* < 0.001). Participants in their 2nd year of study (OR = 0.815, *P* = 0.019) were less likely to feel anxiety than those in their 1st year. Participants majoring in medicine were more likely to report anxiety than those majoring in science (OR = 1.310, *P* = 0.040). University students who wore a mask outside (OR = 0.686, *P* = 0.002), complied with the standard hand-washing technique (OR = 0.701, *P* < 0.001), maintained a one-meter distance in queues (OR = 0.634, *P* < 0.001), and had high psychological resilience scores (OR = 0.980, *P* < 0.001) were less likely to report anxiety over time.

**Table 4:** Longitudinal associations of epidemic preventive behaviors and psychological resilience with anxiety based on GEE.

**Variables**	**Model 1**			**Model 2**			**Model 3**		
	**OR**	**95% CI**	***P***	**OR**	**95% CI**	***P***	**OR**	**95% CI**	***P***
**Gender**									
Male	Ref.			Ref.			Ref.		
Female	0.766	0.684, 0.858	<0.001	0.750	0.668,0.841	<0.001	0.752	0.670,0.845	<0.001
Age	1.054	1.017,1.092	0.004	1.054	1.017,1.093	0.004	1.0270	0.990, 1.066	0.155
**Year of study**									
1st year	Ref.			Ref.			Ref.		
2nd year	0.818	0.702, 0.952	0.010	0.809	0.693,0 0.943	0.007	0.806	0.690,0.940	0.006
3rd year	0.930	0.806,1.073	0.318	0.891	0.771,1.030	0.120	0.888	0.768, 1.027	0.110
4th/5th year	1.221	1.055,1.413	0.007	1.176	1.014,1.364	0.032	1.169	1.007, 1.356	0.040
**Major**									
Science	Ref.			Ref.			Ref.		
Engineering	0.995	0.846,1.171	0.950	1.029	0.873,1.214	0.731	1.018	0.863,1.202	0.829
Agriculture	0.858	0.655,1.125	0.269	0.854	0.650,1.122	0.256	0.857	0.652,1.127	0.270
Liberal arts	1.117	0.956,1.305	0.164	1.163	0.993,1.362	0.062	1.152	0.982, 1.351	0.082
Art	0.858	0.655,1.125	0.269	1.281	1.028,1.596	0.027	1.249	1.000,1.559	0.050
Medicine	1.288	1.007,1.649	0.044	1.321	1.027,1.700	0.030	1.310	1.018,1.686	0.036
**Ethnicity**									
Han	Ref.			Ref.			Ref.		
Minority	1.039	0.793,1.361	0.782	1.097	0.834,1.441	0.509	1.082	0.824, 1.421	0.569
**Student leader**									
Yes	Ref.			Ref.			Ref.		
No	1.053	0.936,1.184	0.390	1.033	0.918,1.163	0.587	1.028	0.912, 1.158	0.651
**Wearing a mask outside**									
No				Ref.			Ref.		
Yes				0.657	0.515,0.838	0.001	0.686	0.538, 0.874	0.002
**Compliance with the standard hand-washing technique**									
No				Ref.			Ref.		
Yes				0.715	0.613,0.834	<0.001	0.701	0.600, 0.817	<0.001
**Maintaining a distance of one meter in queues**									
No				Ref.			Ref.		
Yes				0.644	0.564,0.735	<0.001	0.634	0.555, 0.724	<0.001
Psychological resilience							0.980	0.974, 0.987	<0.001

### 3.4. Association of pandemic preventive behavior and psychological resilience with stress

Table 5 presents the GEE analysis for stress. Model 3 included demographic variables, pandemic preventive behaviors, and psychological resilience. Women were less likely to report stress over time (OR = 0.656, *P* < 0.001). Participants in their 4th or 5th year of study were more likely to report stress over time than those in their first year (OR = 1.385, *P* = 0.011). Those majoring in liberal arts (OR = 1.358, *P* = 0.002), art (OR = 1.373, *P* = 0.021), or medicine (OR = 1.775, *P* ≤ 0.001) were more likely to report stress over time. Additionally, people who complied with the standard hand-washing technique (OR = 0.638, *P* < 0.001), maintained a one-meter distance in queues (OR = 0.638, *P* < 0.001), and had higher psychological resilience scores (OR = 0.976, *P* < 0.001) were less likely to report stress over time.

**Table 5:** Longitudinal associations of epidemic preventive behaviors and psychological resilience with stress based on GEE.

**Variables**	**Model 1**			**Model 2**			**Model 3**		
	**OR**	**95% CI**	***P***	**OR**	**95% CI**	***P***	**OR**	**95% CI**	***P***
**Gender**									
Male	Ref.			Ref.			Ref.		
Female	0.672	0.588, 0.768	<0.001	0.653	0.569, 0.749	<0.001	0.656	0.571, 0.753	<0.001
Age	1.075	1.031, 1.122	0.001	1.077	1.032, 1.124	0.001	1.043	0.998, 1.091	0.061
**Year of study**									
1st year	Ref.			Ref.			Ref.		
2nd year	0.957	0.790, 1.158	0.649	0.954	0.786, 1.158	0.633	0.946	0.779, 1.149	0.575
3rd year	1.111	0.931, 1.325	0.243	1.067	0.892, 1.277	0.476	1.064	0.889, 1.273	0.500
4th/5th year	1.442	1.211, 1.717	<0.001	0.954	0.786, 1.158	0.633	1.385	1.160, 1.652	<0.001
**Major**									
Science	Ref.			Ref.			Ref.		
Engineering	1.113	0.908, 1.363	0.302	1.153	0.939, 1.415	0.173	1.142	0.930, 1.403	0.205
Agriculture	1.039	0.736, 1.465	0.829	1.043	0.738, 1.474	0.810	1.056	0.748, 1.490	0.759
Liberal arts	1.314	1.082, 1.596	0.006	1.372	1.128, 1.670	0.002	1.363	1.119, 1.661	0.002
Art	1.275	0.978, 1.661	0.072	1.414	1.082, 1.848	0.011	1.374	1.048, 1.800	0.021
Medicine	1.735	1.297, 2.322	<0.001	1.778	1.320, 2.394	<0.001	1.775	1.316, 2.394	<0.001
**Ethnicity**									
Han	Ref.			Ref.			Ref.		
Minority	0.856	0.606, 1.209	0.377	0.898	0.633, 1.273	0.546	0.883	0.624, 1.251	0.485
**Student leader**									
Yes	Ref.			Ref.			Ref.		
No	1.084	0.939, 1.251	0.271	1.064	0.920, 1.230	0.401	1.057	0.914, 1.224	0.454
**Wearing a mask outside**									
No				Ref.			Ref.		
Yes				0.775	0.601, 0.999	0.049	0.823	0.638, 1.062	0.134
**Compliance with the standard hand-washing technique**									
No				Ref.			Ref.		
Yes				0.654	0.549, 0.779	<0.001	0.638	0.535, 0.760	<0.001
**Maintaining a distance of one meter in queues**									
No				Ref.			Ref.		
Yes				0.649	0.553, 0.761	<0.001	0.638	0.543, 0.749	<0.001
Psychological resilience							0.976	0.968, 0.983	<0.001

## 4. Discussion

This study investigated the prevalence of depression, anxiety, and stress among Chinese university students during the COVID-19 pandemic while on campus and when returning home for the winter vacation, with an interval of 3 months between these time points. In addition, the longitudinal association of demographic variabless, preventive behaviors, and psychological resilience with depression, anxiety, and stress were examined. To our knowledge, the present study is the first longitudinal study on the associations between mental health, preventive behaviors, and psychological resilience among university students in China during the COVID-19 pandemic. We found that the prevalence of anxiety and stress decreased over time, whereas the rate of depression increased between the time at which university students were on campus and the time at which they returned home for the winter vacation. Additionally, students' psychological resilience decreased longitudinally and the rate of compliance with preventive behaviors increased.

In this longitudinal survey, we found that students' reported rates of depression significantly increased over time (35.2 vs. 36.9%). This may be because COVID-19 may have lasting effects on university students' psychological health (42, 43), and the risk of psychological disorders may increase over time (44). In addition, it is worth noting that, although our investigation was conducted at a time when the pandemic was under control in China, there was still a risk of imported and sporadic cases. Students' psychological status may have fluctuated with the emergence of cases and this may have led to increased rates of depression. The proportion of students reporting anxiety (44.8 vs. 41.2%) and stress (23.0 vs. 19.6%) decreased over time. A possible reason for this decrease is that, while we were conducting the T2 phase of the survey, the Chinese government began to provide COVID-19 vaccines for all citizens, which may have provided psychological comfort to the students (37). Moreover, students returning home for the winter vacation period would feel more secure at home than at school (45).

We also found that students in their 3rd, 4th, or 5th year of study were more likely to report depression and stress than those in their 1st year, which is consistent with previous research (8). This pattern may be because there is more academic pressure on final-year students. They must be prepared for graduation, employment, and internships, but the prevalence of COVID-19 inevitably affects various matters. Additionally, we found that medical students were more likely to report depression, anxiety, and stress. Higher anxiety levels among medical students than among non-medical students were also found during the SARS-CoV-1 outbreak (46). Even in the absence of a pandemic outbreak, studies have shown that medical students report higher levels of psychological distress than their peers of the same age (47). This distress may arise because medical students are more knowledgeable about illnesses than other students (48), which makes them more likely to develop psychological problems.

Our findings suggest that measures taken to prevent the spread of COVID-19 may have had a protective psychological effect on university students. Consistent with previous studies (49), we found that those who wore a mask outside were less likely to report mental health problems. Similarly, those adhering to standard hand-washing techniques and maintaining a one-meter distance in queues were less likely to report mental health problems (50, 51). These findings highlight the importance of promoting preventive behaviors among university students during the COVID-19 pandemic. We also found that more students complied with preventive behaviors in the second survey than in the first, indicating an increased awareness of prevention among students.

The present study also examined the effect of psychological resilience on depression, anxiety, and stress. Consistent with previous studies (23, 24), we found that greater resilience was negatively associated with depression, anxiety, and stress. This may be because students with high psychological resilience can more clearly understand the meaning of positive coping styles (52). Additionally, being better able to handle negative emotions and respond flexibly to external pressures can help individuals to overcome the effects of negative emotions (53). These results suggest that increasing resilience can reduce depression, anxiety, and stress among university students.

## 5. Implications

First, universities should pay attention to the mental health of university students during COVID-19, focusing particularly on the mental health problems of male university students, senior students, and those majoring in liberal arts, arts, and medicine, and should provide timely guidance and support. Second, government departments and universities should continue to publicize and provide guidance on preventative behaviors that can reduce the spread of COVID-19, highlight the importance of protecting university students from the pandemic, and urge students to engage in good preventive behaviors. Finally, universities should encourage students to cultivate psychological resilience through provision of mental health education in order to reduce depression, anxiety, stress, and other psychological problems in the face of major public health emergencies such as COVID-19.

## 6. Limitations

First, the data used in this study were self-reported, which may have resulted in recall bias. Second, other variables that were not measured in this study, such as coping style, length of time spent in isolation, and other potential factors, may have influenced the results. Third, this study used only “yes/no” responses to measure preventive behavior, which may not be a scientific way to capture this variable. Fourth, the ORs for some variables in this study were close to 1, which may be due to the large sample size that made it easy to achieve statistical significance even for small effects. Finally, findings in relation to university students may not apply to the general population, especially to those with lower levels of education. Despite these limitations, this study used a longitudinal design to reveal the psychological changes in university students on campus and during the winter break during the COVID-19 pandemic and to examine the relationships of these changes with preventive behaviors and psychological resilience. In addition, this study had a large number of participants, enabling significantly reliable conclusions to be drawn.

## 7. Conclusion

In the period between two surveys, the prevalence of depression among university students increased, and the prevalence of anxiety and stress decreased. Senior students and medical students were more likely to experience psychological problems. Therefore, these groups of students should receive greater attention. Preventive behaviors were significantly associated with depression, anxiety, and stress; therefore, university students should continue to follow relevant preventive behaviors. Furthermore, psychological resilience was a protective factor against depression, anxiety, and stress, suggesting that improving psychological resilience may help maintain and promote university students' mental health.

## Data availability statement

The raw data supporting the conclusions of this article will be made available by the authors, without undue reservation.

## Ethics statement

The studies involving human participants were reviewed and approved by the Ethical review and ethical approval for the study was obtained from the Institutional Review Board of Public Health and Preventive Medicine at Shandong University (No. LL20200201). Written informed consent was obtained from respondents for inclusion before they participated in the study. The patients/participants provided their written informed consent to participate in this study.

## Author contributions

JZ and FK designed the study and collected the data. HeL performed data analysis and drafted the manuscript. RC, HuL, and XX contributed to reviewing the literature. JX, XJ, MP, and JW reviewed and edited the manuscript. SL, JH, and FK revised it critically for important intellectual content and supervised the writing of the manuscript. All authors read and approved the final manuscript.
